# Cartilage Tissue-Mimetic Pellets with Multifunctional Magnetic Hyaluronic Acid-Graft-Amphiphilic Gelatin Microcapsules for Chondrogenic Stimulation

**DOI:** 10.3390/polym12040785

**Published:** 2020-04-02

**Authors:** Kai-Ting Hou, Ting-Yu Liu, Min-Yu Chiang, Chun-Yu Chen, Shwu-Jen Chang, San-Yuan Chen

**Affiliations:** 1 Department of Materials Science and Engineering, National Chiao Tung University, Hsinchu City 30010, Taiwan; carolhou8@gmail.com (K.-T.H.); minyu28@gmail.com (M.-Y.C.); 2 Department of Materials Engineering, Ming Chi University of Technology, New Taipei City 24301, Taiwan; tyliu@mail.mcut.edu.tw; 3 Department of Biomedical Engineering, I-Shou University, No.8, Yida Rd., Jiaosu Village, Yanchao District, Kaohsiung City 82445, Taiwan; iergy2000@gmail.com; 4 Department of Orthopedics, Kaohsiung Veterans General Hospital, Kaohsiung City 81362, Taiwan; 5 Frontier Research Centre on Fundamental and Applied Sciences of Matters, National Tsing Hua University, Hsinchu City 30013, Taiwan; 6 School of Dentistry, College of Dental Medicine, Kaohsiung Medical University, Kaohsiung City 80708, Taiwan

**Keywords:** cartilage tissue, amphiphilic gelatin microcapsules, tissue-mimetic pellets, magnetic stimulation, CD44 receptor

## Abstract

Articular cartilage defect is a common disorder caused by sustained mechanical stress. Owing to its nature of avascular, cartilage had less reconstruction ability so there is always a need for other repair strategies. In this study, we proposed tissue-mimetic pellets composed of chondrocytes and hyaluronic acid-graft-amphiphilic gelatin microcapsules (HA-AGMCs) to serve as biomimetic chondrocyte extracellular matrix (ECM) environments. The multifunctional HA-AGMC with specific targeting on CD44 receptors provides excellent structural stability and demonstrates high cell viability even in the center of pellets after 14 days culture. Furthermore, with superparamagnetic iron oxide nanoparticles (SPIOs) in the microcapsule shell of HA-AGMCs, it not only showed sound cell guiding ability but also induced two physical stimulations of static magnetic field(S) and magnet-derived shear stress (MF) on chondrogenic regeneration. Cartilage tissue-specific gene expressions of Col II and SOX9 were upregulated in the present of HA-AGMC in the early stage, and HA-AGMC+MF+S held the highest chondrogenic commitments throughout the study. Additionally, cartilage tissue-mimetic pellets with magnetic stimulation can stimulate chondrogenesis and sGAG synthesis.

## 1. Introduction

Articular cartilage disorder most commonly occurs at the conjunction between bones, and the condition is progressively worsened by constant and unavoidable mechanical degeneration. The loss of the normal cartilage tissue will lead to more serious joint diseases like osteoarthritis [1]. Cartilage reconstruction has been a clinical issue for decades because of poor intrinsic ability to repair defects and lacking of specific diagnostic biomarkers. Several types of clinical treatments and pharmacologic therapies are available and effective in reducing pain and increasing mobility of patients [2,3,4]. Nevertheless, those treatments are merely a temporary relief and unable to restore the damaged tissue into its original function [5]. The long-term clinical solutions for cartilage repair are still in demand, and diverse regenerative therapies are consequently being brought to the table.

The hydrogel-based scaffolds have received a big interest as providing a temporary three-dimensional structure [6,7,8,9]. High functional, strong biomechanical properties and long-term biocompatible scaffolds can be made by regulating different materials to promote the cartilage tissue therapies [10,11]. Scaffolds which allow physical supporting often combines with primary chondrocytes therapies to overcome low cell proliferation or dedifferentiation problems. However, these 3D networks still struggle with several challenges such as cell viability, growth factor burst release or low oxygen content. On the other hand, particles serve as a superior medicine approach. Particles have been widely developed for drug delivery systems in tissue engineering applications due to their wide variety and highly regulated potential [12]. In particular, microscale particles were popular among delivering anti-inflammatory drugs or growth factors and hold promise in performing as building block in scaffold for cartilage repair [13,14]. Cruz et al. [15] aggregated gelatin microparticles and chondrocytes into 3D pellets and found higher cell viability over long periods.

As one of the major components in chondrocytes extracellular matrix (ECM), hyaluronic acid (HA) had a linear polysaccharide structure which functioned as a structural element, providing backbones for the component distribution and aggrecan aggregation. HA was also well known to interact with specific receptors including CD44 to regulate signal transduction, cell migration and differentiation [16,17]. Gelatin was a denatured protein from collagen and widely used in drug delivery systems. Because of gelatin’s biocompatibility, low antigenicity, chemical modification possibility and low-cost [18], many studies had encapsulated growth factor in modified gelatin-based particles to stimulate chondrogenesis [8,19,20]. Moreover, gelatin-based microparticles as building blocks for three-dimensional (3D) structures have been wildly used in cartilage engineering [13]. 

In addition to chemical stimulations and highly cartilage-like materials, mechanical forces acting as additional tools were also applied to improve cartilage reconstruction in recent studies to mimic in vivo environments. The proper biophysical stimulations were able to increase proteoglycan synthesis and cartilage-specific gene expression [21,22]. Magnetic nanoparticles are thought of as excellent candidates to apply remote magnetic induced physical stimulation, which also holds the capability of targeting a specific site. Nathalie et al. [23] labeled mesenchymal stem cells (MSC) with magnetic nanoparticles to enhance seeding density and condensation in scaffold. Together with a dynamic bioreactor, MSC differentiation performance was markedly improved. Son et al. [24] exposed bone-marrow-derived human MSC (BM-hMSC) to static magnetic field and magnet-derived shear stress via magnetic nanoparticle. Without hypertrophic effect, biophysical stimulation led BM-hMSC to higher chondrogenic differentiation efficiency.

**Figure 1:**
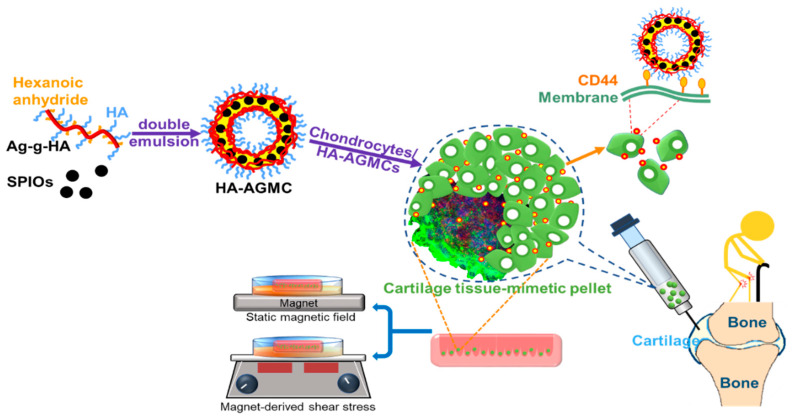
Synthesis of the hyaluronic acid-graft-amphiphilic gelatin microcapsules (HA-AGMCs) structure to fabricate cartilage tissue-mimetic pellets with combined biochemical and biophysical treatments.

We developed a brand-new platform, cartilage tissue-mimetic pellets, to combine biochemical and biophysical treatments to mimic native cartilage tissue. As illustrated in Figure 1, cartilage tissue-mimetic pellets were composed by rabbit primary chondrocytes and hyaluronic acid-graft-amphiphilic gelatin microcapsules (HA-AGMCs). HA polymer chains on the microcapsules surface are expected to expand space between each microcapsule with its highly hydrophilic and polyanionic characteristics. In addition, HA can enhance chondrocytes attachment through CD44 receptors and act stabilize the pellets structure as ECM component at the beginning of formation. We encapsulated superparamagnetic iron oxide nanoparticles (SPIOs) in hydrophobic shells of HA-AGMCs to guide cells and serve physical stimulations by applying static magnetic field and magnet-derived shear stress. The inner hydrophilic space of microcapsules is capable of encapsulating growth factor or biomolecules for cell proliferation or repair. Such HA-AGMC approaches can be used to provide cartilage structure stability, rule biochemical and biophysical stimulations, and thereby promote faster and more complete cartilage reconstruction. 

## 2. Materials and Methods

### 2.1. Materials

Gelatin from porcine skin (type A 300 bloom), hexanoic anhydride, absolute ethanol (99.5%), sodium hydroxide, 2,4,6-Trinitrobenzene Sulfonic Acid (TNBS), 1,2-hexadecanediol (97%), oleic acid (90%), oleylamine (>70%), and iron(III) acetylacetonate (Fe(acac)3) were purchased from Sigma-Aldrich Co. (St. Louis, MO, USA) Hyaluronic acid (Hyalo-Oligo) was purchased from Tannmer Enterprise Co., Ltd. (New Taipei City, Taiwan). N-hydroxysuccinimide (NHS) and 1-ethyl-3-(3-dimethylaminopropyl) carbodiimide hydrochloride (EDC) were purchased from Echo Chemical Co., Ltd. (Miaoli, Taiwan). Platinum^®^ PCR SuperMix and GScript First-Strand Synthesis Kit were purchased from Thermo Fisher Scientific (Waltham, MA, USA).

### 2.2. Synthesis of Hyaluronic Acid-Graft-Amphiphilic Gelatin (AG-g-HA)

Gelatin powder (3 g) was completely dissolved in deionized water (40 mL) at 70 °C with constant stirring for an hour. Eethanol (95%, 30 mL) and hexanoic anhydride (3 mL) were sequentially added dropwise in succession and stirred for 4 h. In this process, the amphiphilic gelatin (AG) form and was tuned to a pH value around 7. The final solution was dialyzed against a mixture of water and ethanol (3:4). AG was collected and dried at 60 °C and then ground into powder with grinding machine. Hyaluronic acid (HA) was grafted to the amino group of the gelatin via EDC/NHS method. HA (1 g) was first dissolved in phosphate buffer solution (40 mL) under stirring. 1-ethyl-3-(-3-dimethylaminopropyl) carbodiimide hydrochloride (EDC, 1 g) and N-hydroxysuccinimide (NHS, 1.9 g) were added to the solution and stirred for one hour at pH 5.5. After the pH was adjusted to 7 with NaOH (10 N), AG (1 g) was added directly and stirred for 2 h. After the reaction, AG-g-HA formed and dialyzed (Spectra/Por, MWCO = 20000) against water before dried by a freeze vacuum dryer. AG-g-HA was analyzed by nuclear magnetic resonance spectroscopy (VARIAN, UNIYTINOVA 500 NMR) with 600-MHz 1H-NMR. Each copolymer (10 wt %) was dissolved in D2 O to obtain 1 H NMR and 13 C NMR spectra.

### 2.3. Quantification of HA Grafting Rate

We used 2,4,6-Trinitrobenzene Sulfonic Acid (TNBS) reagent to determine the content of free amino groups. TNBS (0.025% *w*/*v*) was dissolved in sodium hydrogen carbonate solution (4%) serving as reaction buffer. Gelatin, AG, and AG-g-HA were dissolved in dH2O (1 mL), and mixed with reaction buffer (0.5 mL) separately. Calibration curve was made by mixing Lysine (2–20 μg in 1 mL dH2O) with reaction buffer (0.5 mL). After incubating at 37 °C for two hours, sodium dodecyl sulfate (SDS, 10% *w*/*v*) and 1 N HCl were added to each sample. An absorbance peak at 336 nm was measured with UV-vis spectroscopy (UV-vis, Evolution 300, Thermo, Waltham, MA, USA). The number of free amines contained in each polymer was quantified by correlating with the calibration curve.

### 2.4. Synthesis of Superparamagnetic Iron Oxide Nanoparticles (SPIOs)

The synthesis of SPIOs (6~8 nm) were prepared according to Sun et al [25]. In brief, Fe(acac)3 (2 mmol), 1,2–hexadecanediod (10 mmol), oleic acid (6 mmol), and olecylamine (6 mmol) were mixed in benzyl ether (20 mL) in three-necked bottle. Under nitrogen atmosphere, the mixture was refluxed at 100 °C for 30 min, and then sequentially heated to 200 °C for 1 h and 300 °C for 30 min. After cooling to room temperature, the product was collected by centrifugation at 6000 rpm for 5 min and washed with ethanol three times. The black-brown SPIOs were stored in ethanol at 4 °C.

### 2.5. Preparation of Hyaluronic Acid-Graft-Amphiphilic Gelatin Microcapsule (HA-AGMC)

The amphiphilicity and the self-assembly property of AG together with SPIOs have been investigated by Li et al. and Chiang et al. [19,26]. HA-AGMCs were prepared through a simple double emulsion process. First, AG-g-HA (80 mg) was added to deionized water (1.6 mL) and NaOH solution (1.6 mL, 0.1 N). The solution (0.6 mL) with hydrophobic SPIO (10 mg) in chloroform (1 mL) were emulsified to obtain a water-in-oil (W/O) emulsion. AG-g-HA solution (2.4 mL) was added to proceed the secondary emulsion to form the W/O/W emulsion. Afterward, the organic solvent was removed by rotary evaporator at 33 °C. The final HA-AGMC products were washed 3 times and re-dispersed with deionized water.

### 2.6. Characterization of HA-AGMCs

The size and zeta potential of HA-AGMCs were characterized by dynamic light scattering (Beckman Coulter Delsa™ Nano C particle analyzer, Beckman Coulter, Brea, CA, USA). The morphology of HA-AGMCs were characterized by scanning electron microscope (SEM, JEOL-6700, JEOL, Tokyo, Japan) and transmission electron microscope (TEM, JEM-2100, JEOL, Tokyo, Japan). The samples of SEM were prepared by placing HA-AGMCs solution on a silicon wafer and drying in a vacuum desiccator. TEM samples were prepared by laying HA-AGMCs solution on a carbon coated grid, followed by removing the excess liquid on the grid, and drying in a vacuum desiccator.

### 2.7. Loading Efficiency of SPIOs

SPIOs content was measured using UV-vis spectroscopy (UV-vis, Evolution 300, Thermo). Calibration curve was made by dissolving SPIOs (10 mg) and AG-g-HA (75 mg) in HCl (0.5 N). From UV-vis spectroscopy, the absorbance of the solution was measured at 363 nm. HA-AGMC was also diluted with HCl (0.5 N) to calculate the corresponding loaded SPIOs concentration. 

### 2.8. Chondrocyte Isolation and Culture

Chondrocytes were isolated from the articular cartilage of New Zealand White rabbits (0.4–0.8 kg). All procedures conformed to the guidelines of the Institute of Animal Care and Use Committee of I-Shou University in Taiwan. All the surgical instruments were sterilized before use. After rinsing thighbones with phosphate-buffered saline (PBS) two times, cartilage tissue from the joint was dissected and cut into pieces of approximately 1 × 1 mm^2^ samples. These samples were digested with protease (20 mg in 10 mL DMEM/F-12) for 2 h and transferred to collagenase (20 mg in 10 mL DMEM/F-12) for another 3 h. The cell suspension was centrifuged at 2000 rpm for 5 min and resuspended in DMEM/F12 medium with 10% FBS in 75T culture flask. 

Chondrocytes were seeded in monolayer and used within two passages. The cells were cultured in DMEM/F12 medium with 10% FBS in a 5% CO_2_ incubator at 37 °C. The medium was renewed every 2 days and cells were passaged once it reached confluence.

### 2.9. Cell cytotoxicity Test

Cell cytotoxicity was carried out using the MTS method. In brief, chondrocytes were harvested from culture flask and seeded in a 24-well tissue culture plates at a density of 5 × 104 cells/well. HA-AGMCs were added to the cell culture in different concentrations (0, 0.05, 0.11, 0.21, 0.43, 0.85, 1.7, 3.4, 6.8, 13.6 mg mL^−1^). After 1, 2, or 4 days incubation, culture medium was replaced with DMEM/F-12 containing 10% MTS and reacted for 2 h. Each medium was collected and centrifuged at 6000 rpm for 5 min to remove HA-AGMCs. The absorbance of supernatant was monitored by ELISA reader at wavelength 490 nm. The experiment was done in triplicate.

### 2.10. 3D culture Methods

The experiment method of agarose hydrogels followed the protocol of 3D Petri dish molds [27]. Briefly, agarose powder (1 g) was dissolved in PBS and then pipetted into micro-mold without creating any bubble in proper temperature. After solidification, the agarose hydrogels were placed in culture medium to equilibrate for at least 15 min, and then transferred to fresh medium for further use or storage. All steps were in sterile conditions. Cells at a final density of 2.56 × 105 cells/190 µL were seeded in each agarose hydrogel placing in 6 well plate and waited 10 min to settle cells. Finally, we added additional medium to the plate (2.5 mL/well).

### 2.11. Cell proliferation and Cell Compatibility Analysis

MTS assay was performed to assess the proliferation of chondrocytes. In short, fresh media containing different concentrations of HA-AGMCs (42.5, 85, 170, 340, and 680 μg mL^−1^) were mixed with chondrocytes before seeding in each agarose hydrogel placing in plate and waited 10 min to settle cells. Finally, additional medium was added to cover the hydrogel. After incubation for various time period, cell pellets were collected and counted before adding 10% MTS medium. Each medium was collected after 3 h reaction and the optical density was monitored by ELISA reader at wavelength 490 nm. The experiment was run in four times. Cell compatibility was also evaluated by Live/Dead assay. After days of incubation, cell pellets were collected and washed with PBS buffer. Staining reagent Calcein AM and Ethidium homodimer-1 in PBS covered the pellets at 37 °C for 30 min. The samples were observed under confocal microscopy. The experiment was done in triplicate. The fluorescence intensity in each pellet was measured by ImageJ and further analyzed by unpaired *t*-test.

### 2.12. HA-AGMCs Cellular Attachment Efficiency

To measure the precise amount of HA-AGMCs attached on chondrocytes, we examined the element content of Fe by inductively coupled plasma mass spectrometry (ICP-MS). To prepare the sample, we collected 1 mL medium in vials after chondrocytes mixing with various amounts of HA-AGMCs and seeded on hydrogel overnight. Hydrochloric acid (12 M), which can totally corrode the cell sample and redox iron in HA-AGMC, was mixed in all the samples. The content of Fe element was quantified by ICP-MS.

### 2.13. RNA Isolation, cDNA Synthesis and Quantitative PCR Analysis

The procedure of RNA isolation was bottomed on Molecular Research Center, Inc. In short, chondrocytes pellets were washed with PBS buffer and added 1mL of RNAzol^®^ RT reagent to lyse cells. Subsequently, added DEPC-treated water (0.4 mL) to lysate and shook the mixture vigorously for 15 s. Centrifuged samples for 15 min at 13,500 rpm at 4 °C after storing for 15 min. The RNA remained soluble in the supernatant. Each of the supernatant (1 mL) was transferred to a new tube and precipitated by mixing with 0.4 mL of 75% ethanol. The samples were centrifuged for 10 min at 12000 rpm at 4 °C after stored for 15 min. RNA precipitated and formed a white pellet at the bottom of the tube. Next, we washed the RNA twice with 75% ethanol to remove the ethanol and dissolve the RNA precipitation pellet with DEPC-treated water.

Complementary DNA was performed for reverse transcription using the GScript First-Strand Synthesis Kit (GeneDireX). Specific cDNA was amplified by PCR using Platinum^®^ Blue PCR SuperMix. The protocol of experiment method followed Invitrogen manufacturer. cDNA samples were mixed up with Platinum^®^ Blue PCR SuperMix and forward/reverse primer. The PCR amplification condition was illustrated as follows: heating up to 95 °C for 5 min, denaturing the DNA at 95°C for 30 s, annealing with the primer at 55 °C for 30 s, extending the length of product at 72 °C for 30 s, and finally cycled for 35 times. PCR cycle ended at 72 °C for 3 min and cooled down to 4 °C. The samples were stored at −20 °C and electrophoresis experiment ran at voltage 80 V, 35 min. The experiment was also conducted in three times.

### 2.14. Alcian Blue Staining

Alcian blue solution was prepared beforehand by dissolving Alcian blue 8GX powder in 40% acetic acid and 60% ethanol mixture solvent in 1 wt % concentration, and the solution was stored at 4 °C. At the end of the culture period, cell pellets were washed with PBS buffer and fixed with 4 % formaldehyde for 1 h. The fixed-cell samples were washed with PBS buffer and then incubated with Alcian blue solution overnight. The stained samples were washed with PBS buffer and mounted by mounting solution. The observation was using optical microscope. The experiment was run in triplicate and the results were statistically analyzed using two-way ANOVA Dunnett’s multiple comparisons test comparing with the control group (* *P* < 0.05, ** *P* < 0.01, *** *P* < 0.001, and **** *P* < 0.0001).

### 2.15. Static Magnetic Field (MF) and Magnet-Derived Shear Stress (S) Stimulations

A cylinder-shaped neodymium magnet with a magnetic field of 0.22 T was used to static magnetic field induction [24,28]. On the other hand, a magnetic stirrer of 50 rpm was applied for magnet-derived shear stress stimulation on the cell-pellets. For combined stimulation, cell-pellets were first placed on the top of the magnet and then transferred to the magnetic stirrer on pellets in consecutive order following static magnetic field and/or magnet-derived shear stress for 1 h one day for five consecutive days

## 3. Results and Discussion

### 3.1. Synthesis and Characterization of AG-g-HA

**Figure 2:**
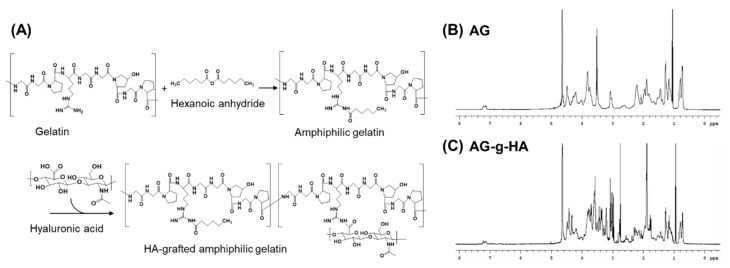
(**A**) The reaction scheme for the synthesis of hyaluronic acid-grafted amphiphilic gelatin (AG-g-HA); ^1^H NMR spectrum of macromolecules in D2O for (**B**) amphiphilic gelatin (AG) and (**C**) AG-g-HA. As shown, the NMR spectra at 3.5, 1.2, 1.9 and around 3.5 ppm demonstrated the successful synthesis of AG-g-HA.

Figure 2A illustrated the reaction scheme for the synthesis of Hyaluronic acid-grafted amphiphilic gelatin (AG-*g*-HA). As a widely used biopolymer, gelatin is composed of a series of amino acids, serving as hydrophilic backbone and modified by hexanoic anhydride to gain amphiphilic characteristic. HA was conjugated on gelatin’s Arginine (Arg) part by the well-known EDC/NHS method. The chemical signals of the gelatin (Figure S1 and Table S1) from peak 1 to peak 11 assigned to the protons on the primitive gelatin macromolecules [29]. According to the NMR spectra in Figure 2B, chemical signals of amphiphilic gelatin (AG) at peak 3.5 ppm and peak 1.2 ppm were the protons from the hexanoyl group. The disappearance of peak at 2.9 ppm which was referred to the primary amino group of the gelatin confirmed that amino group, arginine, was partially substituted to hexanoyl group forming AG. The α-carbonyl group (COCαH3) at 1.9 ppm and glucose group between 3.2 and 3.9 ppm gave the cue of glycosylation (Figure 2C). TNBS is a well-known reagent specific for primary amino groups, which can be quantified and measured at absorbance 335 nm [30]. The substitution rate of the hexanoyl group was measured to be 58.2%. The second HA conjugation rate was 4.0% (Figure S2). The results demonstrated that there were finally 37.8% free amino groups remaining in the arginine and lysine of the gelatin where hexanoyl group and HA substitution were at 58.2% and 4.0%, respectively.

### 3.2. Characterization of HA-AGMCs and Cartilage Tissue-Mimetic Pellets

**Figure 3:**
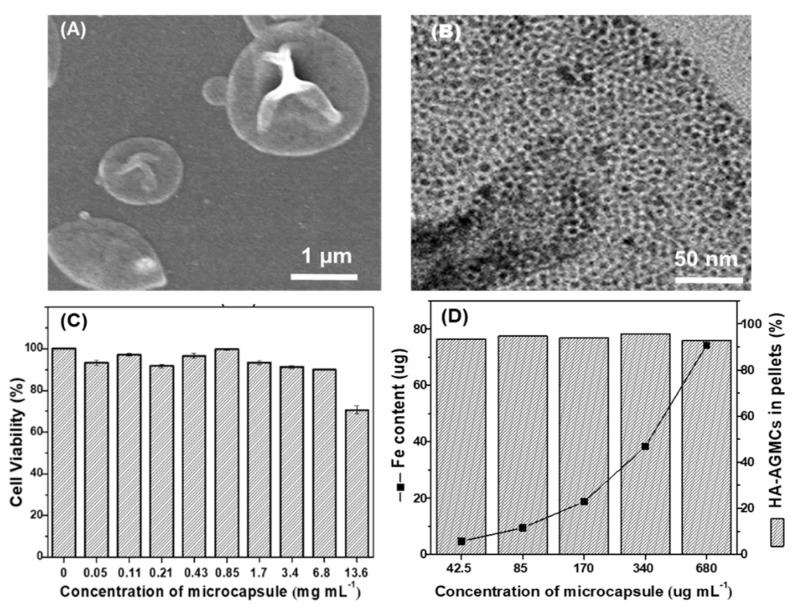
Morphological appearance and characterization of the HA-AGMCs. (**A**) SEM and (**B**) TEM images. (**C**) Biocompatibility of HA-AGMCs to chondrocytes after 24 h incubation (n = 3). (**D**) Attachment efficiency of HA-AGMCs to chondrocytes in different concentrations of HA-AGMC. The results demonstrated HA-AGMCs have a spherical-shaped bilayers structure with average diameter of 1.2 µm and negligible cytotoxicity to chondrocytes.

Hyaluronic acid-graft-amphiphilic gelatin microcapsule (HA-AGMC) was synthesized via double emulsification using SPIOs and AG-g-HA. The diameter and zeta potential of HA-AGMCs were measured by dynamic light scattering (DLS) (Table S2). HA-AGMC exhibited an average size of 1.24 ± 0.1 µm in diameter with an excellent monodispersity (Figure S3). The surface charge was approximately −16 mV caused by the replacement of positively charged amino groups to hexanoyl group and HA. HA-AGMCs demonstrated the appearance of deflated balloons structure in scanning electron microscope (SEM) images (Figure 3A). The round hollow morphology can be further confirmed by transmission electron microscopy (TEM) image in Figure 3B where the little dark spots of SPIOs were clearly observed in the shell of microcapsules. The loading efficiency of SPIOs in HA-AGMCs was 92.2% determined by ultraviolet-visible spectroscopy (UV-vis).

The biocompatibility of HA-AGMC with rabbit primary chondrocytes was investigated by MTS assay (Figure 3C). The cells were treated with different concentrations of HA-AGMC from 0.05 to 13.6 mg mL^−1^. HA-AGMC illustrated outstanding biocompatibility at high concentration in which chondrocytes viability was higher than 84% after 24 h co-culture with HA-AGMC under 6.8 mg mL^−1^.

In this study, hyaluronic acid (HA) on HA-AGMC surface was designed to help sticking chondrocytes and microcapsules together. The CD44 antigen was the main receptor for hyaluronic acid, which was responsible for cell proliferation, differentiation and migration [16,17,31]. Hyaluronic acid conjugated on the surface of microcapsule was capable of binding to the chondrocytes due to CD44/HA receptors and serving as a backbone to recruit proteoglycans and glycoproteins into extracellular matrix structures [32]. Attachment efficiency of HA-AGMCs to chondrocytes was used to evaluate the formation of cartilage tissue-mimetic pellets by co-culturing chondrocytes with HA-AGMCs on non-adhesive micro-molds (Figure 3D). The Fe amount was also quantified by using ICP-MS and increased with HA-AGMCs concentration. The attachment efficiency was over 90% in each HA-AGMCs group with the microcapsule concentration of 42.5 to 680 µg mL^−1^. The result demonstrated that mostly HA-AGMCs can easily attach to chondrocytes and form pellets together. 

**Figure 4:**
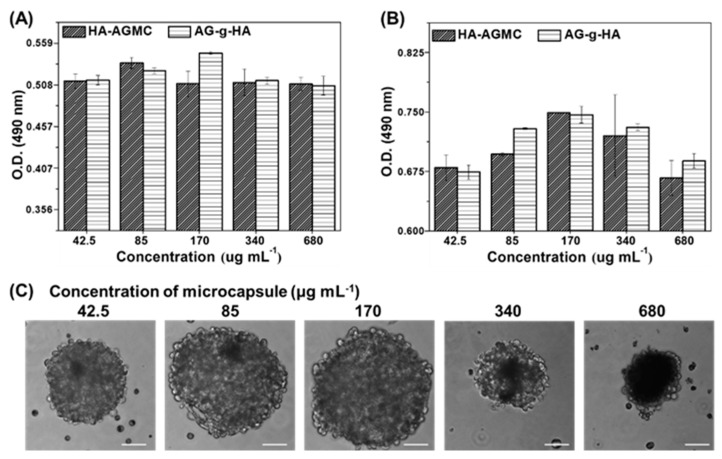
Cartilage tissue-mimetic pellets proliferation and morphology. The relative cell proliferation after culturing chondrocytes with HA-AGMCs microcapsule or AG-g-HA at different concentrations (from 42.5 to 680 µg mL^−1^) at (**A**) 7 days and (**B**) 14 days (n = 4). (**C**) The cell pellet morphologies after culturing 14 days showed that HA-AGMCs at the concentration of 170 µg mL^−1^ had the biggest pellet size of 200 μm. Scale bar = 50 µm.

We fabricated cartilage tissue-mimetic pellets with different concentrations of microcapsule HA-AGMCs and also used macromolecule AG-g-HA as control group. The number of viable chondrocytes and the morphology of cartilage tissue-mimetic pellets were examined by MTS assay and optical microscope at day 7 and 14 respectively. HA-AGMCs and AG-g-HA showed little difference in optical density (O.D.) over the cultural period at different concentrations from 42.5 to 680 µg mL^−1^ (in AG-g-HA concentration) (Figure 4A,B). The results illustrated that the HA-AGMCs microcapsules containing SPIOs do not lower the number of chondrocytes. Of note, after 14 days of culture, HA-AGMCs at the concentration of 170 µg mL^−1^ had the relative highest cell number correlated to the optical microscope images (Figure 4A). The morphology of pellets in different HA-AGMCs concentrations after culturing 14 days was shown (Figure 4C). Pellets in each group showed uniform size and rounded shape, and more microcapsules made the pellets looked darker responding to more SPIOs. During the culture period, a scatter of cell debris was found in the group with 680 mL^−1^ HA-AGMCs, which resulted in a smaller size of pellet. In contrast, HA-AGMCs at the concentration of 170 µg mL^−1^ had the biggest pellet size of 200 μm. Therefore, the HA-AGMCs at 170 µg mL^−1^ was selected for the following all experiments.

### 3.3. Cartilage Tissue-Mimetic Pellets Live/Dead Assay

**Figure 5:**
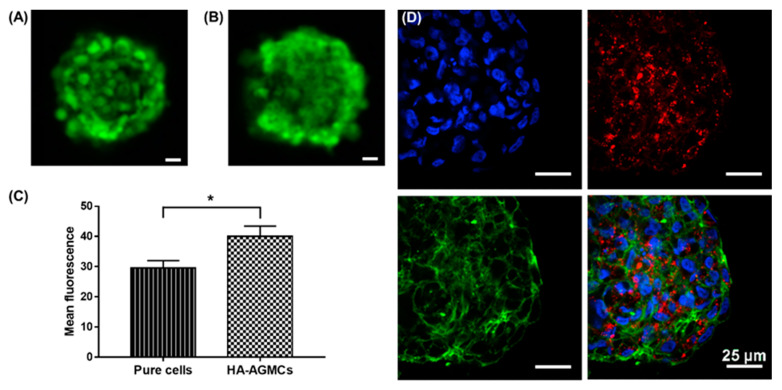
Confocal fluorescent image of cartilage tissue-mimetic pellets after 14 days culture. Live/Dead fluorescent image of (**A**) pure cells control group and (**B**) cells with HA-AGMCs group. Calcein AM (green), Ethidium homodimer-1 (red). (**C**) The fluorescence intensity in each pellet was measured by ImageJ and further analyzed by unpaired *t*-test (*P* = 0.0123, n = 3). (**D**) Quantum dots were encapsulated in the shell of HA-AGMCs and used to visualize the localization of cartilage tissue-mimetic pellets consisting of DAPI (blue), actin (green), HA-AGMC (red quantum dots). As shown, chondrocytes remained viable in HA-AGMCs group and had stronger connection to ECM. Scale bar = 25 m.

We tested long-term cell viability with Live/Dead assay to confirm cell condition, and to investigate whether HA-AGMCs had any influence on the cartilage tissue-mimetic pellets. In 3D culture, the supplement of oxygen was mainly controlled by diffusion, consequently resulting in oxygen gradient. Oxygen tension was often found in the center of the 3D structure and also dependent on the number of cells. The nutrient and oxygen supply in the central of pellets would be increasingly inadequate during the culture period [33,34]. In this study, fluorescence images showed after 14 days, the vast majority of cells remain viable in both pure cells control group and HA-AGMCs group (Figure 5A,B). The mean fluorescence intensity in each HA-AGMCs pellet showed more cells and higher viability compared to cell only group as shown in Figure 5C. In comparison to clear boundary between each cell in control group (Figure 5A), HA-AGMC group showed blurry edge, indicating cell pellets in HA-AGMC group had stronger connection to ECM (Figure 5B). This further confirmed that HA-AGMC are able to promote the connection between cells and construct chondrocytes into a compact pellet. To observe the localization of HA-AGMC, quantum dots were dissolved in chloroform with SPIOs and encapsulated in the shell of HA-AGMC. The confocal fluorescent images showed cooperative formation of HA-AGMC and chondrocytes at 14 days (Figure 5D), HA-AGMCs still remained in the pellets and scattered uniformly in the ball-shaped pellets. Our studies demonstrated that microcapsule HA-AGMCs can maintain great cell compatibility and viability in cartilage tissue-mimetic pellets.

### 3.4. Gene expression of Cartilage Tissue-Mimetic Pellets under Physical Stimulations

**Figure 6:**
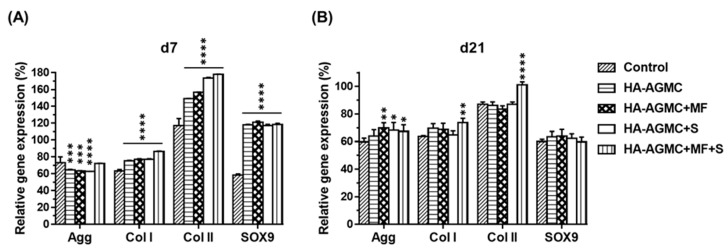
Gene expressions of cartilage tissue-mimetic pellets under static magnetic field (MF) and magnet-derived shear stress (S) stimulations at (**A**) day 7 and (**B**) day 21. The results were statistically analyzed using two-way ANOVA Dunnett’s multiple comparisons test comparing with the control group (* *P* < 0.05, ** *P* < 0.01, *** *P* < 0.001, and **** *P* < 0.0001). HA-AGMC helped chondrocytes maintain cartilage tissue-specific gene at the beginning of pellets forming and applying both MF and S hold the gene expression better.

HA-AGMC was designed as a multifunctional platform to guide the cartilage tissue-mimetic pellets and serve physical stimulations. Static magnetic field (MF) and magnet-derived shear stress (S) were applied on the HA-AGMCs one hour each day for five consecutive days. In addition to mimic cartilage ECM environment, joint movements mechanical forces also played a crucial part in growth and development of articular cartilage tissue. It was found that stem cell performed better properties when cells grew in scaffold under external mechanical stimulations [23,35,36]. More recently, magnetic nanoparticles captured more attention to bioreactor as they were capable of providing different mechanical forces and were more suitable for applying loading to improve cell condensation and scaffold seeding efficiency [37,38,39]. We performed biophysical stimulations via SPIOs and analyzed the gene expression of Aggrecan (Agg), collagen type I (Col I), collagen type II (Col II), and Sox9 with polymerase chain reaction (PCR) after 7 and 21 days of culture. The primer sequences were shown in Table S3 and housekeeping genes GAPDH were used in comparison with the samples. After 7 days culture period, the presence of HA-AGMCs significantly increased Col I, Col II and Sox9 gene expression compared to control group. HA-AGMC+MF+S and control group showed similar Agg gene expression (Figure 6A). The gene expression of Col II and Agg gave us an indication of chondrocytes’ functionality as they attributed to the secretion of ECM to stabilize the structure of ECM [40]. It is worth noting that Sox9 gene regulated chondrogenesis and chondrocyte proliferation [41], and the expressions of Sox9 were dramatically upregulated 2-fold in all HA-AGMCs-added groups after 7 days. Taking up-regulation of Agg, Col II, and Sox9 contrasting to Col I gene expression altogether, HA-AGMC-treated group demonstrated a slightly better chondrocyte expansion. The HA-AGMC with physical stimulation-treated group showed significant Agg gene expression compared to control group after 21 days culture period (Figure 6B). Furthermore, gene expression of both Col I and Col II revealed significant difference between HA-AGMC+MF+S and control group. Taking the results together, the application of HA-AGMCs and biophysical stimulation did not generate dedifferentiation effect on chondrocytes due to the relatively lower expression of Col I than any other group during culture period. Furthermore, HA-AGMC+MF+S group performed the highest Col II gene expression level throughout the culture period, despite of HA-AGMC+S with sound Col II gene expression at the early stage. These data indicated that HA-AGMC can help chondrocytes present in cartilage tissue-specific gene at the beginning of pellets formation, and somewhat maintain functional gene expression better after applying both static magnetic field and magnet-derived shear stress. However, further studies are needed to optimize the mimic native cartilage environment in terms of biophysical parameters. 

### 3.5. Synthesis of Sulfated Glycosaminoglycan under Physical Stimulations 

**Figure 7:**
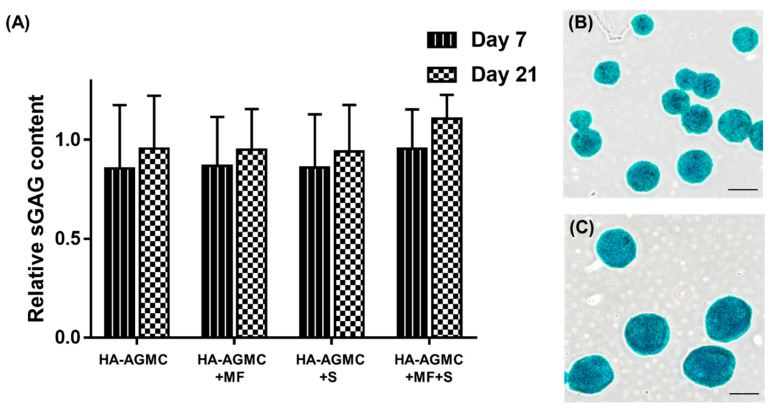
The cartilage tissue-mimetic pellets productivity of sGAG. (**A**) Blyscan assay detected sGAG content in culture medium under different stimulations at 7 and 21 day (n = 3). Alcian blue staining demonstrated the secretion of sGAG after 21 days culture of (**B**) control and (**C**) HA-AGMCs. scale bars = 200 μm.

The synthesis of sulfated glycosaminoglycan (sGAG) is one important index of chondrocyte biochemical function [41,42], and the diminishing presence of sGAG indicated a tendency to de-differentiate into fibrochondrocytes [43]. The cartilage tissue-mimetic pellets productivity of sGAG under physical simulations was examined by Blyscan assay (Figure 7A). The sGAG production increased with incubation time. Physical stimulations further enhanced cartilage tissue-specific ECM production, and HA-AGMC+MF+S group exhibited the greatest sGAG secretion in medium after 21 days culture (Figure 7B). Alcian blue staining results further demonstrated that both and HA-AGMCs group had high content of sGAG excretion in comparison with pure cells control group in Figure 7C where, staining results were not further quantified because of the SPIOs interference.

### 3.6. Histological Analysis

**Figure 8:**
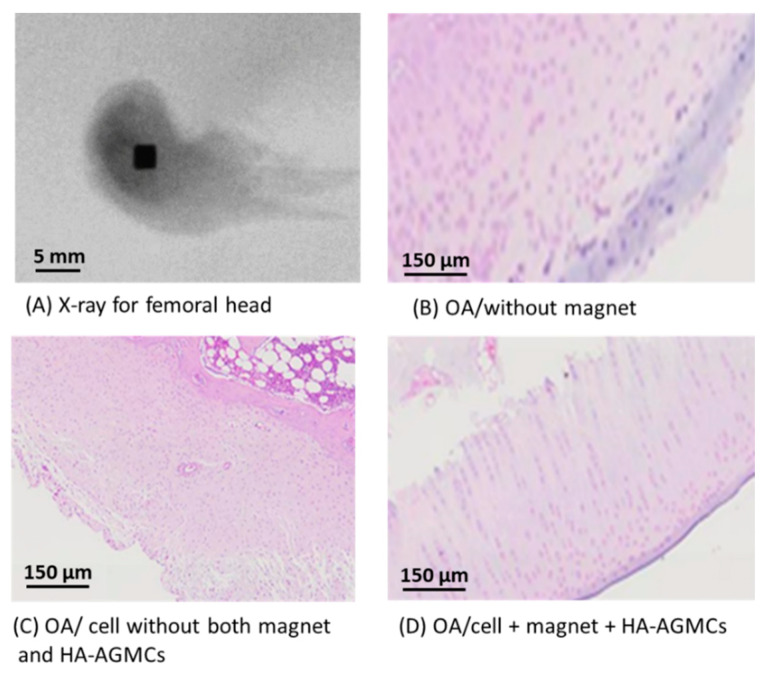
(**A**) Half of the rabbit’s femoral head implanted with a magnet as a source of magnetic force. H&E stain of the cartilage tissue treated with various groups of cells/magnet/HA-AGMCs for 4 weeks in (**B**–**D**).

The cell pellets with HA-AGMCs at the concentration of 170 µg mL^−1^ was used for animal implant experiments to make in vivo chondrogenic analysis in an osteoarthritic mode. Osteoarthritis was surgically induced by anterior cruciate ligament transection (ACLT) and partial medial meniscectomy on one knee of male New Zealand rabbits. Half of the rabbit’s femoral head are implanted with a magnet as a source of magnetic force as shown in Figure 8A. In this in-vivo experiment, the HA-AGMCs are pre-cultured with chondrocytes before implantation into rabbit OA knee. Subsequently, after 6 weeks of surgery, the rabbits received implants of pellets containing either only cells or HA-AGMCs with cells or nothing as control groups. 

After 4 weeks of implantation, the preliminary results revealed that control groups without HA-AGMCs displayed irregular layer of cartilage surface and degeneration of the cartilage tissue (as illustrated in Figure 8B). In addition, the abnormal growth and disorder of cartilage tissues were observed in pure cell without magnet and HA-AGMCs group shown in Figure 8C. More importantly, a newly formed tissue is evident in the group treated with a combination of chondrocytes with A-AGMCs and magnet in Figure 8D, which demonstrated that the cells treated with HA-AGMCs and magnetic force can improve the retention and biofunctionality of transplanted chondrocytes to form ordering arrangement in cartilage matrix, which is very important for cartilage repair. However, the comprehensive investigation about chondrogenic analysis and cartilage repair is in subsequent progress, which will be reported in the future.

## 4. Conclusions

In summary, cartilage tissue-mimetic pellets have considerable advantages in over articular cartilage disorder obstacles. In this study, Am-HA-gelatin has been synthesized by modifying primary amino group on gelatin with hexanoic anhydride to obtain the amphiphilic property and then grafting hyaluronic acid on the amphiphilic gelatin which is capable to form simple core-shell hollow structure. The multifunctional HA-AGMC with specific targeting on CD44 receptors provided cartilage tissue-mimetic pellets with high structure stability and remained high cell viability even in the center of pellets after 14 days culture. Packing with superparamagnetic iron oxide nanoparticles (SPIOs) in the HA-AGMC microcapsule, the pellets with the magnetic HA-AGMCs demonstrated the combination of static magnetic field and magnet-derived shear stress can exhibit the highest cartilage tissue-specific gene expression. Our preliminary results have showed that the cells treated with HA-AGMCs and magnetic force exhibit the better growth and ordering of chondrocytes.

## Supplementary Materials

The following are available online at https://www.mdpi.com/2073-4360/12/4/785/s1, Figure S1: ^1^H NMR spectrum of molecules in D2O for gelatin., Figure S2: Quantification of gelatin-based molecules with TNBS. (A) Absorption spectra of primary amino group of the gelatin reacted with TNBS at different concentrations. (B) Characterization of different degrees of substitution for the gelatin-based molecules., Figure S3: Particle size distribution profile of HA-AGMCs, Table S1: The protons on the primitive gelatin exhibited chemical shift signals from peak 1 to peak 12., Table S2: Characterization of the HA-AGMCs, Table S3: List of genes and the primers used for PCR in chondrocytes.
