# Supplementary material for: Cartilage Tissue-Mimetic Pellets with Multifunctional Magnetic Hyaluronic Acid-Graft-Amphiphilic Gelatin Microcapsules for Chondrogenic Stimulation
Source: Polymers (Basel). 2020 Apr 2;12(4):785. doi: 10.3390/polym12040785 (PMC7240739; doi:10.3390/polym12040785)
Supplement: Supplementary file 1 [file polymers-12-00785-s001.pdf]

Supplementary

for

Cartilage tissue-mimetic pellets with multifunctional magnetic hyaluronic acid-graft-amphiphilic gelatin microcapsules for chondrogenic stimulation

Note-1 ^{1}H NMR spectrum of molecules in D_2O for gelatin

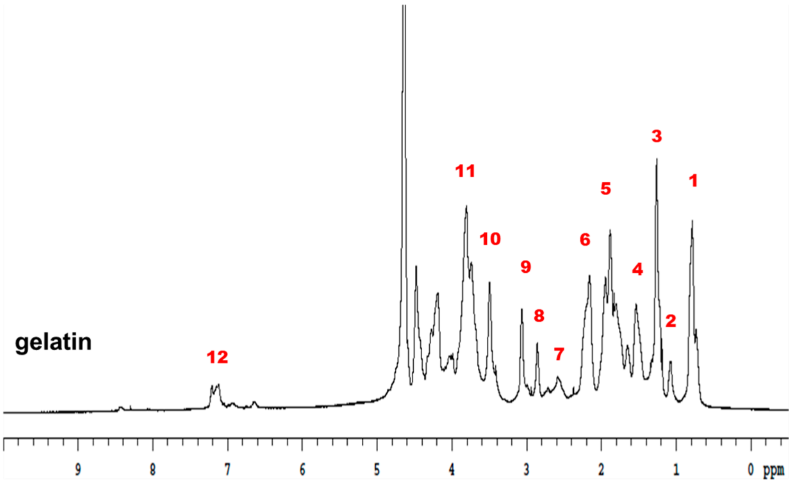

Figure S1. ^{1}H NMR spectrum of molecules in D_2O for gelatin.

Table S1. The protons on the primitive gelatin exhibited chemical shift signals from peak 1 to peak 12.

No. of line	p.p.m	Amino acids	Type of protons
1	0.8	Val, Leu, I-Leu	γ- and δ-CH_3
2	1.1	Thre	γ-CH_3
3	1.3	Ala	CH_3
4	1.5	Arg,<br>Lys	β- and γ-CH_2<br>β-, γ- and δ-CH_2
5	1.9	Pro,<br>Meth	β-, γ- and δ-CH_2<br>β-CH_2 and CH_3
6	2.2	Glu,<br>Hypro	β- and γ-CH_2<br>β-CH_2
7	2.6	Asp	β-CH_2
8	2.9	Lys	ε-CH_2
9	3.1	Arg,<br>Hys	δ-CH_2<br>β-CH_2
10	3.5	Pro	δ-CH_2
11	3.8	Gly, Hypro	α-CH_2<br>γ-CH
12	7.1-7.2	Phen, Tyr	Aromatic C-2, C-6

Note-2. Quantification of gelatin-based molecules with TNBS

A

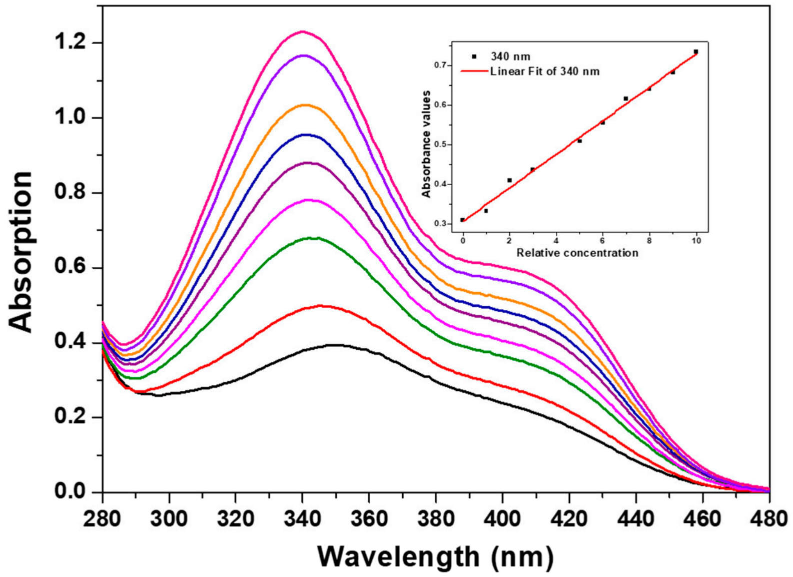

B

	NH_2 group (%)	conversion (%)	Second conversion (%)
**gelatin**	100.0	0.0	
**Am-gelatin**	41.8	58.2	
**AG-g-HA**	37.8	62.2	4.0

Figure S2. Quantification of gelatin-based molecules with TNBS. (A) Absorption spectra of primary amino group of gelatin reacted with TNBS at different concentrations. (B) Characterization of different degrees of substitution for the gelatin-based molecules.

Note-3. Characterization of the HA-AGMCs

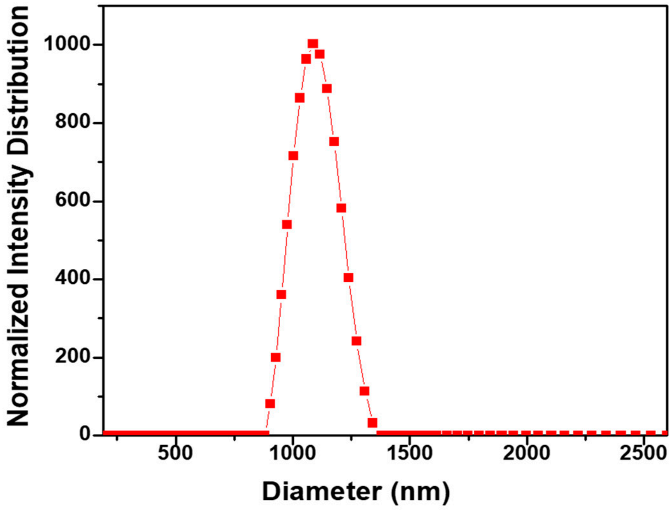

Figure S3. Particle size distribution profile of HA-AGMCs

Table S2. Characterization of the HA-AGMCs

Microcapsule	Size (d. µm)	PDI	Zeta potential (mV)	SPIOs loading efficiency (%)
HA-AGMC	1235.2 ± 97.8	0.2 ± 0.0	-15.8 ± 1.4	92.2

Note-4. Genes and the primers used for PCR in chondrocytes

Table S3. List of genes and the primers used for PCR in chondrocytes.

GAPDH	forward	AGGTCATCCACGACCACTTC
	Reverse	GTGAGTTTCCCGTTCAGCTC
Aggrecan (Acan)	Forward	GACTCTTTCACATGCTTATGCCTTCC
	Reverse	GTGACGATGCTGCTCAGGTGTG
Collagen type I	forward	TAAGAGCTCCAAGGCCAAGA
	Reverse	TGTTCTGAGAGGCGTGATTG
Collagen type II	Forward	CAACAACCAGATCGAGAGCA
	Reverse	GCTCCACCAGTTCTTCTTGG
SOX9	Forward	TTCATGAAGATGACCGACGA
	Reverse	GTCCAGTCGTAGCCCTTGAG
